# New Insight into Sperm Capacitation: A Novel Mechanism of 17β-Estradiol Signalling

**DOI:** 10.3390/ijms19124011

**Published:** 2018-12-12

**Authors:** Tereza Bosakova, Antonin Tockstein, Natasa Sebkova, Ondrej Simonik, Hana Adamusova, Jana Albrechtova, Tomas Albrecht, Zuzana Bosakova, Katerina Dvorakova-Hortova

**Affiliations:** 1 Department of Analytical Chemistry, Faculty of Science, Charles University, Albertov 2030, 128 43 Prague, Czech Republic; terezabosakova@seznam.cz (T.B.); atockstein@seznam.cz (A.T.); hana.adamusova@gmail.com (H.A.); 2 Laboratory of Reproductive Biology, Institute of Biotechnology CAS, v.v.i., BIOCEV, Prumyslova 595, 252 50 Vestec, Czech Republic; Natasa.Sebkova@ibt.cas.cz (N.S.); simoniko@af.czu.cz (O.S.); 3 Department of Veterinary Sciences, Faculty of Agrobiology, Food and Natural Resources Czech University of Life Sciences Prague, Kamycka 129, 165 00 Prague, Czech Republic; 4 Department of Zoology, Faculty of Science, Charles University, Vinicna 7, 128 44 Prague, Czech Republic; jana.brehova@seznam.cz (J.A.); albrecht@ivb.cz (T.A.); 5 Institute of Vertebrate Biology, v.v.i., Czech Academy of Sciences, Kvetna 8, 603 65 Brno, Czech Republic

**Keywords:** 17β-estradiol, sperm, capacitation, acrosome reaction, kinetics, autocatalysis, HPLC MS/MS, CASA

## Abstract

17β-estradiol (estradiol) is a natural estrogen regulating reproduction including sperm and egg development, sperm maturation—called capacitation—and sperm–egg communication. High doses can increase germ cell apoptosis and decrease sperm count. Our aim was to answer the biological relevance of estradiol in sperm capacitation and its effect on motility and acrosome reaction to quantify its interaction with estrogen receptors and propose a model of estradiol action during capacitation using kinetic analysis. Estradiol increased protein tyrosine phosphorylation, elevated rate of spontaneous acrosome reaction, and altered motility parameters measured Hamilton-Thorne Computer Assisted Semen Analyzer (CASA) in capacitating sperm. To monitor time and concentration dependent binding dynamics of extracellular estradiol, high-performance liquid chromatography with tandem mass spectrometry was used to measure sperm response and data was subjected to kinetic analysis. The kinetic model of estradiol action during sperm maturation shows that estradiol adsorption onto a plasma membrane surface is controlled by Langmuir isotherm. After, when estradiol passes into the cytoplasm, it forms an unstable adduct with cytoplasmic receptors, which display a signalling autocatalytic pattern. This autocatalytic reaction suggests crosstalk between receptor and non-receptor pathways utilized by sperm prior to fertilization.

## 1. Introduction

In order for mammalian sperm to fertilize, it must undergo a series of maturation events in the female reproductive tract, called capacitation [1]. In general, capacitation involves membrane rearrangement, cholesterol efflux, activation of specific signal transduction pathways leading to protein tyrosine phosphorylation (TyrP), and cytoskeleton rearrangements [2,3,4]. As much as capacitation-related increase of TyrP has been postulated to be a key capacitation marker [1,3], a study [5] showed that this may not be an essential event in mouse sperm, and lack of TyrP can be bypassed in vivo by an as yet unknown mechanism. Therefore, it is important to evaluate the status of capacitation by other parameters, such as motility and sperm ability to undergo acrosome reaction (AR). Hamilton-Thorne Computer-Aided Sperm Analysis (CASA) is generally used for the monitoring of sperm motility and provides a set of standard kinematic parameters that aids with the identification of changes in trajectories and flagellar beating patterns connected with hyperactivation as an important part of sperm capacitation [6,7]. In vivo capacitation occurs mainly in the uterus and oviducts, facilitated by substances in the female genital tract including estrogen. It has been demonstrated that endocrine disruptors, including estrogen, significantly modulate capacitation in vitro and can also increase its speed [8,9,10,11,12]. Estradiol and its analogues induced not only capacitation but also non-induced (spontaneous) AR [13]. Progesterone-induced AR [9,13], progesterone-enhanced sperm hyperactivation [14,15,16], and progesterone-increased TyrP [14,15] are all suppressed by estradiol.

Estrogen cell signalling is a crucial event, and both general and sperm specific mechanisms are still not understood. An interaction between the most influential of estrogen, estradiol and its estrogen receptors (ERs), has been described through nuclear receptors (nER) and membrane (mER) and cytoplasmic (cER) receptors [17,18]. These receptors might be either of the same composition, just translocated from the nucleus to the membrane [19,20], or they may represent a novel kind of ERs [21,22,23]. In addition to the estrogen-based receptors, the ability of non-ER membrane-associated proteins to bind and transfer estradiol across the plasma membrane (PM) has been described [24]. On the top of the transportation receptors, estradiol is able to penetrate and pass through the PM without any help [25,26]. The complexity of the system is stressed by two routes of estradiol action: the slow genomic and the rapid non-genomic routes. The generally excepted model is built on the binding of the intracytoplasmic estradiol to the well-described and understood nER, targeting DNA sequences known as estrogen response elements (EREs) [27] or DNA-binding transcription factors [28,29,30], both leading to transcriptional activation of the associated genes [31,32]. The genomic pathway requires cell transcriptional activity and time, none of which these mature spermatozoa possess. In this case, the non-genomic pathway remains the only option for sperm in order to regulate the signalling pathways that are crucial for the capacitation processes. For obvious reasons, it is difficult to study capacitation in vivo; therefore, it is crucial to understand precise in vitro conditions, including dynamics, speed, and amount of exogenous estrogen that is bound to sperm receptors during capacitation. Importantly, there has been reported genetic variation between mouse strains, which play a role in fertility success and sperm quality [33,34,35]. Therefore, strain specific differences also need to be addressed.

The mathematical interpretations and general quantified process dynamics bring another perspective to help explain this crucial biological event. In a previous study [36], the mathematical theory for the mechanism when sperm maturation ability is amended by external stimuli [37] was proposed. It was shown that the chemical kinetics can be applied to sperm in the role of reactant and that kinetic analysis could be a useful tool for monitoring and predicting the specific molecular mechanisms involved in certain biological signalling pathways. Kinetic analysis data can serve as a tool for predicting the general hormone-receptor mechanism and provide new insights into the selected biological reaction. At the moment, the scientific community knowledge has extended to the description of individual signalling scenarios in both somatic and male germinal cells, but the mechanism of the action remains unknown.

The objectives of this study were as follows: (1) to clarify the effect of estradiol on sperm capacitation, motility, and acrosome reaction; (2) to develop a sensitive analytical high-performance liquid chromatographic method with tandem mass spectrometric detection (HPLC-MS/MS) for the determination of total unbound estradiol concentration in the sperm capacitation in vitro; and (3) to reveal whether the concentration of estradiol versus the time of ongoing capacitation fits an equation or a system of equations of chemical kinetics. By applying kinetic analysis to our data obtained by HPLC-MS/MS, and measuring the estradiol dynamics during mouse sperm capacitation, we propose a unique type of autocatalytic estradiol signalling, which could unify all the estradiol actions into one complex event.

## 2. Results

### 2.1. Monitoring of Estradiol Concentration during Capacitation In Vitro by HPLC

**Figure 1:**
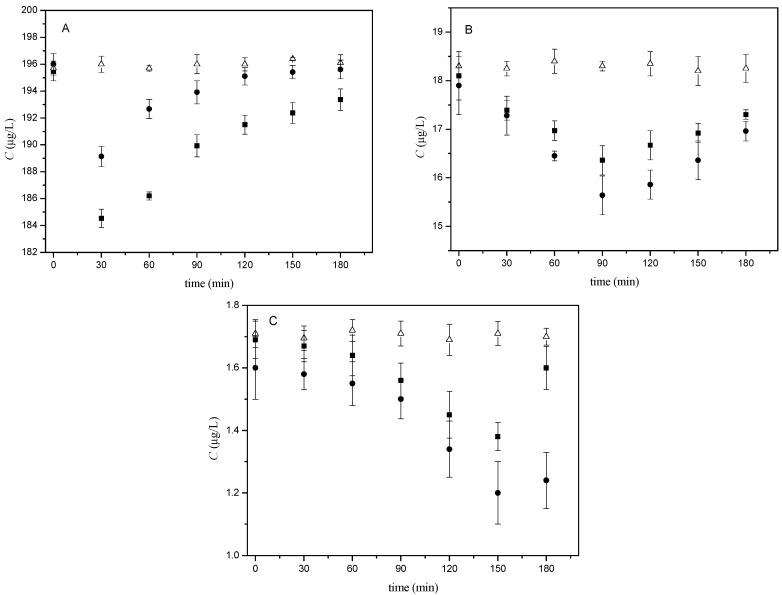
Dependencies of the concentration of total unbound estradiol on the time of mouse sperm capacitation in vitro. The tested concentrations of estradiol in M2 medium were (**A**) 200 μg/L, (**B**) 20 μg/L, and (**C**) 2 μg/L. Each experiment was carried out for samples with added mouse sperm (solid circles for BALB/c sperm, solid squares for C57BL/6Nvel sperm) and for reference samples without sperm (blanks, open triangles). The samples were prepared in three parallel sets, where each set represented sperm collecting from one individual; each sample was measured in five replicates; experimental conditions: 50/50 (*v*/*v*) acetonitrile (ACN)/H_2_O, both containing 0.1% HCOOH, measured in multiple reaction monitoring (MRM) mode for transition 255.2 to 158.9; error bars were calculated using the standard deviations (*n*′ = 3). For details, see Section 4.2 and Section 4.3 and Table A1 in Appendix B (Supplementary Materials).

One of the key compounds of the culture media used for in vitro capacitation is bovine serum albumin (BSA), which is responsible for the transfer of cholesterol and phospholipids from the plasma membrane bilayer and is essential for sperm capacitation [1,3,38,39]. In order to monitor the effects of estradiol on sperm capacitation in vitro, it was essential to determine how much estradiol, added into commercial culture media for in vitro sperm capacitation and fertilization (M2 medium, Sigma-Aldrich, Darmstadt, Germany), is available for sperm as the process of binding of estradiol to BSA is known [40]. This was determined for all three tested estradiol concentrations 200, 20, and 2 μg/L (730, 73, and 7.3 nM, respectively) after one hour of in vitro sperm incubation in M2 medium at 37 °C and 5% CO_2_. These calculated values were taken as the initial concentrations at capacitation time 0 and the dependencies of total unbound estradiol concentration on capacitation time were measured. The process of capacitation was monitored in the time range of 0 to 180 min with a 30-min interval between sample collection. Extending the time to over 180 min had no physiological relevance, as the capacitation of mouse sperm in vivo is achieved within 90 min (180 min at the latest). In order to cover genetic variation between mouse strains, we performed all the experiments for a laboratory inbred albino (BALB/c) and black (C57BL/6Nvel) house mouse strains and compared the results (Figure 1).

As can be seen from Figure 1A–C, all the tested estradiol concentrations in blank samples remain practically constant (open triangles) during capacitation. For samples with the addition of sperm (solid circles and squares), similar trends were obtained for all the tested concentrations (200, 20, and 2 µg/L) and both spermatozoa of BALB/c or C57BL/6Nvel mouse laboratory strains. In general, the concentration of total unbound estradiol decreases to reach its minimum and then increases again. However, the position of the minimum differs for the individual tested estradiol concentrations. For the starting concentration 200 µg/L (Figure 1A) the straightforward decrease occurred at the beginning of capacitation time within 0–30 min. The drop of 20 µg/L at the starting concentration can be observed later at 60–90 min (Figure 1B) and the dependence obtained for starting concentration 2 µg/L exhibited its minimum between 150–180 min (Figure 1C). Only slightly different extents of decline were observed between two different strain origins of spermatozoa within the individually tested estradiol concentration (solid circles for BALB/c sperm versus solid squares for C57BL/6Nvel sperm).

Analogical measurements were run under non-capacitating conditions using a medium without BSA. No differences between the samples and blanks were observed for the dependencies of total unbound estradiol concentrations on incubation time (0–180 min) and the estradiol concentrations remained constant up to 180 min.

### 2.2. Kinetic Analysis

Given that kinetic analysis has already been successfully used for analysis of data describing the reduced capacitation ability of sperm [36], it was also used to analyse the reaction of sperm with estradiol. The course of a sperm response on the added estradiol (Figure 1) indicated an autocatalytic character with a formation of an unstable adduct followed by its decomposition. Therefore, these results were subjected to kinetic analysis. For a better comparison of the measured time-dependent concentrations of total unbound estradiol (*C*), the relative concentrations of total unbound estradiol (*B*) were introduced defined as *B*_t_ = *C*_t_/*C*_t=0_, where *B*_t_ is the relative concentration of total unbound estradiol calculated for the capacitation times (0–180 min), *C*_t_ is the concentration of total unbound estradiol measured at capacitation times (30–180 min) and *C*_t=0_ is the concentration of total unbound estradiol measured at capacitation time 0 min. Calculated *B*_t_ values for BALB/c and C57BL/6Nvel mouse strains are given in Table 1.

**Figure 2:**
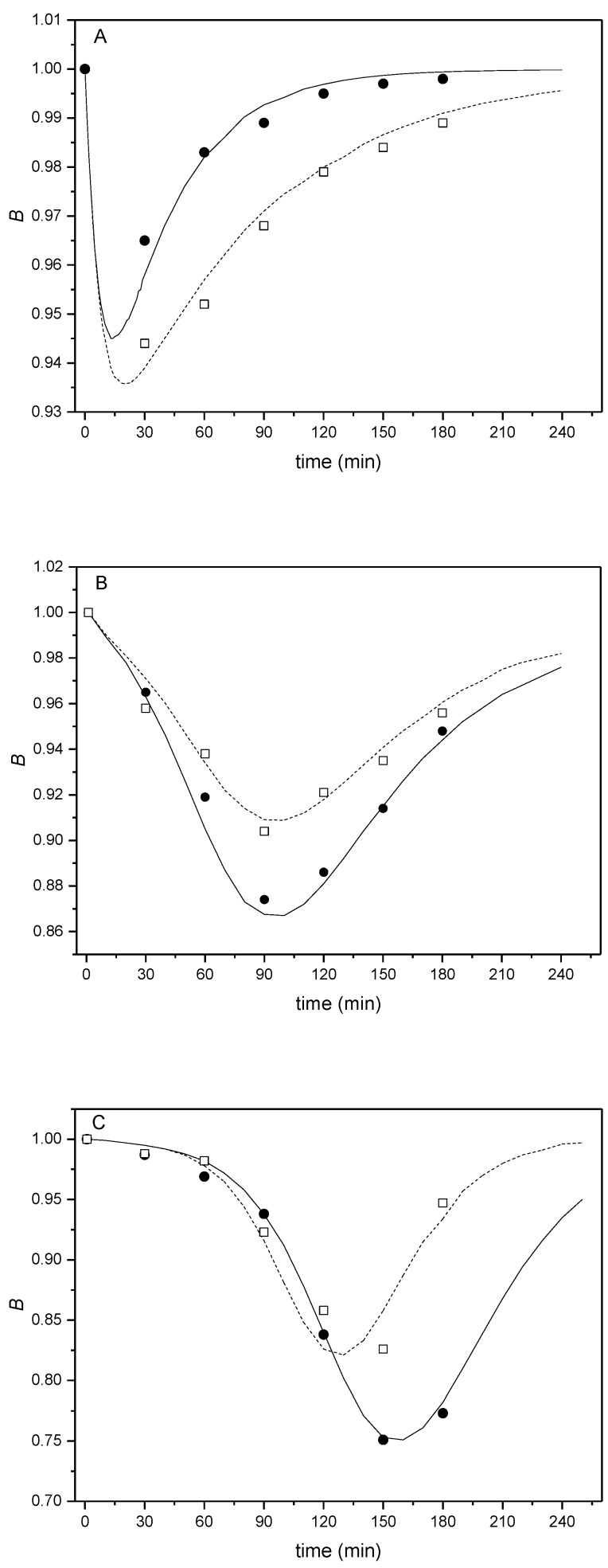
Theoretical shape of the *B*(*t*) curves (solid line for BALB/c, dashed line for C57BL/6Nvel) obtained by integration of kinetic equations (details in Appendix A in Supplementary Materials) for the selected dilution *D*, molar ratio *n*, and optimized values of *K*_2_ and *K*_3_ (Table 2) with designation of the points obtained in the experiment (solid circles for the BALB/c experiments, and solid squares for the C57BL/6Nvel experiments) for estradiol concentrations; (**A**) 200 μg/L, (**B**) 20 μg/L, and (**C**) 2 μg/L.

The suggested kinetic schema was described by kinetic products and rate constants in the form of rate equations. Relative values for individual variables were introduced. The system of differential equations was solved by numerical integration with the simultaneous optimization of the rate constants used to calculate the theoretical *B*_t_ values. Using MATLAB software, optimal parameters (overall rate constants *K*_2_, *K*_3_ and molar ratio *n*) were determined by searching the minimum of absolute values of the difference between theoretical and experimental *B*_t_ values (Table 2). These values were then used for the design of the theoretical *B*(*t*) curves. The fit of the theoretical *B*(*t*) curves and experimentally obtained data is shown in Figure 2. For a detailed description of mathematical procedure, see Appendix A (Supplementary Materials).

Referring to Figure 2, the experimental points fit the theoretical *B*(*t*) curves well for both tested mouse strains. Firstly, the tangent slope of these curves increases, which means that the reaction between estradiol and cytoplasmic receptors accelerates and the formation of their adduct (*E*2/*cER*) takes place autocatalytically. The minima appeared at times *t*_min_ through the formation and subsequent decomposition of the adduct. An important feature of the *B*(*t*) curves is a considerable shift in *t*_min_ with decreasing estradiol concentration (200 → 2 µg/L). The kinetic analysis showed that the initial adsorption at specific membrane centres (Langmuir adsorption isotherm) is a prerequisite for an autocatalytic reaction associated with gradual increase in membrane fluidity in the formation of an adduct (signalling process). The position of *t*_min_ in relation to the time at 10-fold and 100-fold dilution of the highest employed concentrations of estradiol (200 µg/L), i.e., 1/10 or 1/100 of the added amount of estradiol, indicates that the reaction of estradiol with the sperm receptors does not have an integral order because the position of *t*_min_ would not change for first-order but would change 10-fold for a second-order reaction (Appendix A in Supplementary Materials).

A slight shift was observed between the theoretical curves reflecting differences in estradiol binding dynamics between two mouse inbreed strains, BALB/c and C57BL/6Nvel, supporting the strain/sub-strain-specific phenotypic responses for mice of different genetic backgrounds, which is also reflected in the fertilizing ability of sperm [33]. However, the theoretical curves *B*(*t*) fit the experimental points for both tested types of mice with the same outcome, which supports the evidence of finding the theoretical mechanism, which fits to differences between mouse strains. The shift in experimental data may be related to the fact that C57BL/6Nvel mice have lower fertility efficiency [34] and sperm motility parameters, such as reaching the hyperactivated stage the sperm capacitation [35].

### 2.3. Capacitation Status Monitored by TyrP

**Figure 3:**
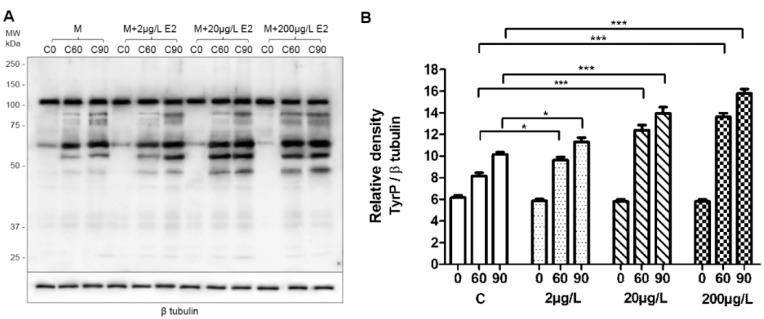
Time-dependent sperm protein TyrP in presence of estradiol (E2) under capacitating conditions as assessed by Western blot analysis and relative densitometry. (**A**) Control samples: sperm capacitating in commercial M2 medium (M); M + E2: the medium enriched by E2 in different concentrations (2, 20, and 200 μg/L). In contrast with freshly released epididymal sperm (t0), protein TyrP increased during sperm capacitation (t60, t90) with a maximum value at 90 min (t90). The protein TyrP was stronger when E2 was present. Increasing concentrations of E2 lead to stronger protein TyrP. β-tubulin was used as the loading control. The samples contained a protein equivalent of 106 cells. Five experiments per group were prepared; the representative results are shown. (**B**) Densitometry analysis of TyrP protein levels in sperm of control samples and sperm incubated in M2 medium enriched by E2. The densitometry analysis indicates relative levels of TyrP revealed a time-dependent and significant increase. Error bars indicate the SD. The statistical significance of the differences among protein TyrP abundances in different groups is indicated by asterisks (* *p* < 0.05, *** *p* ≤ 0.001).

**Figure 4:**
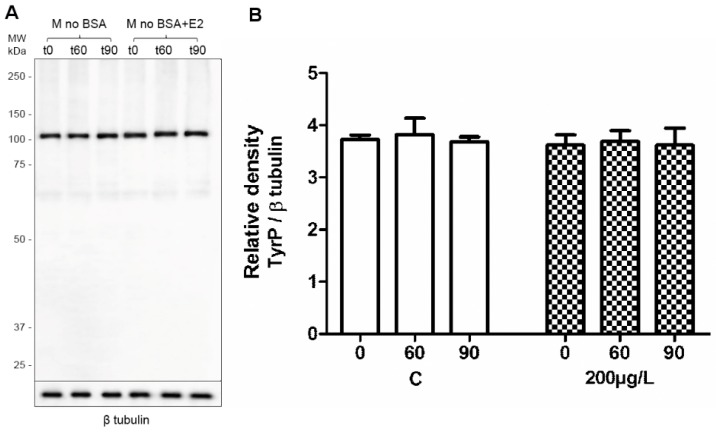
Time-dependent sperm protein TyrP in presence of estradiol (E2) under non-capacitating conditions by Western blot analysis and relative densitometry. (**A**) Control samples: sperm capacitating in commercial M2 medium (M); M + E2: the medium enriched by E2 in different concentrations (2, 20, and 200 μg/L); M with no BSA; and M with no BSA + E2: sperm left in the medium without BSA or without BSA with the addition of E2 (200 μg/L) for 60 and 90 min. Increasing protein TyrP was not observed for any time (t60, t90) or experimental conditions. All incubation times and conditions indicate the same patterns that were identical to the non-capacitating sperm pattern (t0). β-tubulin was used as the loading control. The samples contained a protein equivalent of 106 cells. Five experiments per group were prepared, representative result shown. (**B**) Densitometry analysis of TyrP protein levels in sperm of control samples and sperm incubated in M2 medium enriched by E2 (200 μg/L). The densitometry analysis indicates the same patterns in all the incubation times and conditions. Error bars indicate the SD. There was no statistical significance among protein TyrP abundances in different groups.

During capacitation, signalling pathways are activated resulting in protein phosphorylation, especially on tyrosine (Tyr) residues, which are the key marker of successfully ongoing capacitation process [3]. In order to monitor capacitation status and see whether estradiol kinetics changes under conditions that do not support capacitation, a series of experiments monitoring time-dependent (0, 60, and 90 min) sperm protein TyrP in absence (control) or presence of estradiol under capacitating (Figure 3) and non-capacitating (Figure 4) conditions were run. The presence of BSA in the medium is essential for binding cholesterol from the sperm plasma membrane, which consequently results in the signalling pathway activation and leads to the initiation of sperm capacitation. This cascade of events does not happen in the absence of BSA [1,3,41]. We monitored the time-dependent sperm protein TyrP using Western blot (WB) protein analysis (Figure 3 and Figure 4) and immunofluorescence detection (Figure A1, Appendix B in Supplementary Materials).

The results of WB analysis showed differences in the amount of protein TyrP between the control samples (Figure 3) and sperm that were incubated in BSA-absent medium (Figure 4). The results show that, during sperm capacitation, a typical increase in the number of proteins that were phosphorylated on tyrosine residues occurred, and TyrP was elevated when estradiol was added (Figure 3). These results correspond to previous findings [11,12]. TyrP no longer increased after 90 min of capacitation and remained steady thereafter (results not shown). In the absence of BSA in the medium (its precise composition as M2 except for BSA) (Figure 4), the protein detection did not show any increase in TyrP, and its level corresponded to the control time 0, when sperm capacitation was not initiated (Figure 3). We did not detect any rescue effect by estradiol addition. TyrP did not occur in that group either (Figure 4).

Sperm head protein TyrP plays a crucial role in sperm-*zona pellucida* (an extracellular matrix of the egg) recognition and its level is much lower (up to 15%) compared to complete sperm tail protein TyrP [42]. In order to evaluate TyrP sperm head proteins, sperm smears were assessed by immunofluorescence labelling. The graph showing the percentage of sperm head TyrP during sperm capacitation (Figure A1, Appendix B in Supplementary Materials) indicates an increasing level of TyrP reaching the highest values (approx. 15%) in 90 min of capacitation in the control. When estradiol was added at the beginning of sperm capacitation, the number of positive sperm heads raised to 20% within 90 min, which correlated to previously published work [11]. In contrast (Figure A1, Appendix B in Supplementary Materials), TyrP labelling was significantly lower (approx. 3%) in all samples where sperm were incubated in the medium without BSA, and the addition of estradiol did not provoke the sperm head protein TyrP. The TyrP values of the groups in absence of BSA statistically correspond to the non-capacitated sperm.

The results from WB agreed with those of fluorescence, even though WB reflects the level of whole sperm protein TyrP compared to fluorescence analysis, which only focused on sperm head status. The analysis proved that, in the medium without BSA, sperm capacitation does not occur, not even in the presence of estradiol, which, in capacitation conditions, increase the protein TyrP and do not promote the capacitation in non-capacitating conditions. For detailed information on methodology, please see Appendix A in Supplementary Materials.

### 2.4. Sperm Motility and Hyperactivation Measured by CASA

Linear mixed effects model with lateral head displacement (ALH) as the dependent variable indicated that estradiol concentrations affected strains in a different way (interaction term *concentration x strain* was significant: χ^2^ = 11.29, ΔDf = 3, *p* = 0.01). Strains were therefore analyzed separately. ALH was, to some extent, affected by estradiol in C57BL/6Nvel (effect of estradiol concentration: χ^2^ = 8.28, ΔDf = 3, *p* = 0.041) and increased compared to control in estradiol concentration 20 µg/L by 0.73 ± 0.25 (SE) µm. There was no effect of estradiol concentrations on ALH in BALB/c (χ^2^ = 4.95, ΔDf = 3, *p* = 0.19) (Table A2, Appendix B in Supplementary Materials).

In curvilinear velocity (VLC), the interaction term *concentration x strain* was also significant (χ^2^ = 10.676, ΔDf = 3, *p* = 0.014). Estradiol concentrations had a clear effect on sperm motility in BALB/c (effect of estradiol concentration: χ^2^ = 16.35, ΔDf = 3, *p* < 0.001). VLC was reduced compared to the control, particularly at estradiol concentrations of 2 µg/L (reduction 12.78 ± 3.45 (SE) µm/s) and 20 µg/L (reduction 7.98 ± 3.45 (SE) µm/s). In contrast, the presence of estradiol had a weak negative, but insignificant, effect on C57BL/6Nvel (effect of estradiol concentration: χ^2^ = 1.57, ΔDf = 3, *p* = 0.67; Table A2, Appendix B in Supplementary Materials). For detailed information on methodology, please see Appendix A in Supplementary Materials.

### 2.5. Acrosome Reaction

**Figure 5:**
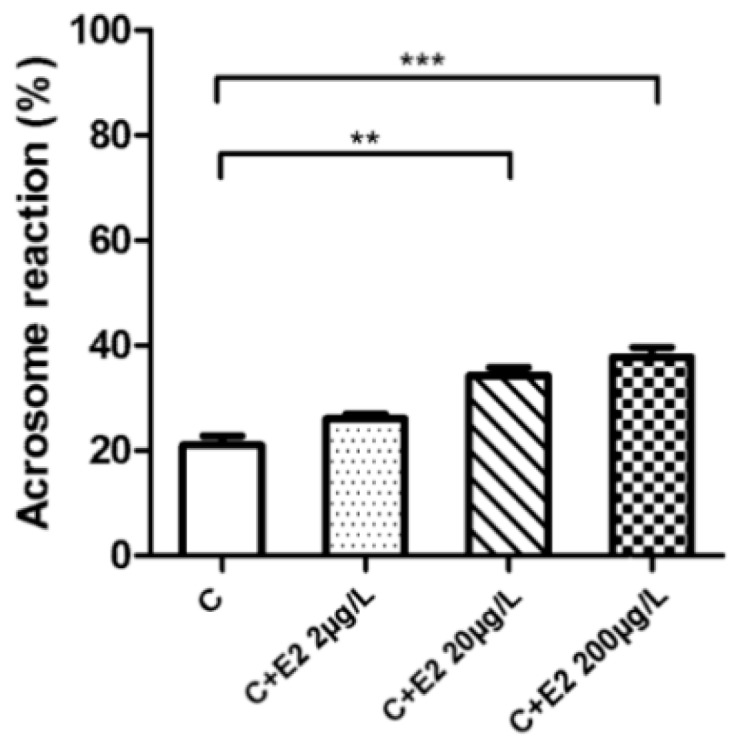
Acrosome reaction during 90 min capacitation (C) in presence of estradiol (E2). Sperm were capacitated in presence of E2 (C + E2 2; 20 and 200 μg/L) or without E2 (control, C). The rate of acrosome reaction (AR) was monitored by peanut agglutinin lectin (PNA). A dose-dependent increase in spontaneous AR during 90 min capacitation was demonstrated. A total of 200 cells were counted in six individual samples. The differences in percentage distribution of sperm with intact acrosome and acrosome reacted sperm was statistically analyzed. Error bars represent the SD. (** *p* < 0.01, *** *p* < 0.001).

**Figure 6:**
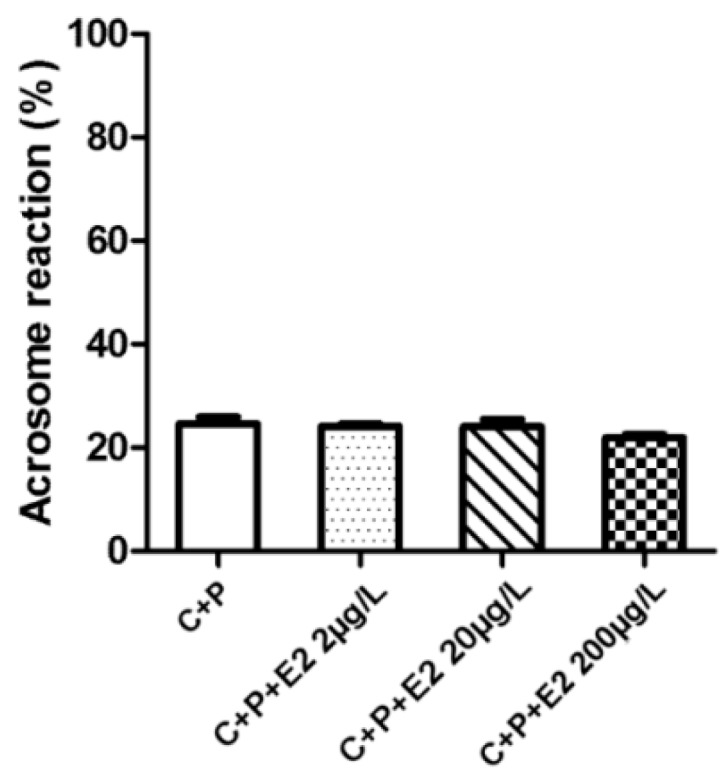
Acrosome reaction during 90 min capacitation (C) in simultaneous presence of estradiol (E2) and progesterone (P). Sperm were capacitated in capacitating medium in presence of progesterone (10 μM, 3144 µg/L) with E2 (C + P + E2: 2, 20, and 200 μg/L) or without E2 (control, C + P). A total of 200 cells were counted in six individual samples. No differences were detected in contrast with control samples. The AR rate was the same for all different experimental E2 concentrations. Error bars indicate the SD. There was no statistical significance among sperm incubations with different E2 concentrations.

**Figure 7:**
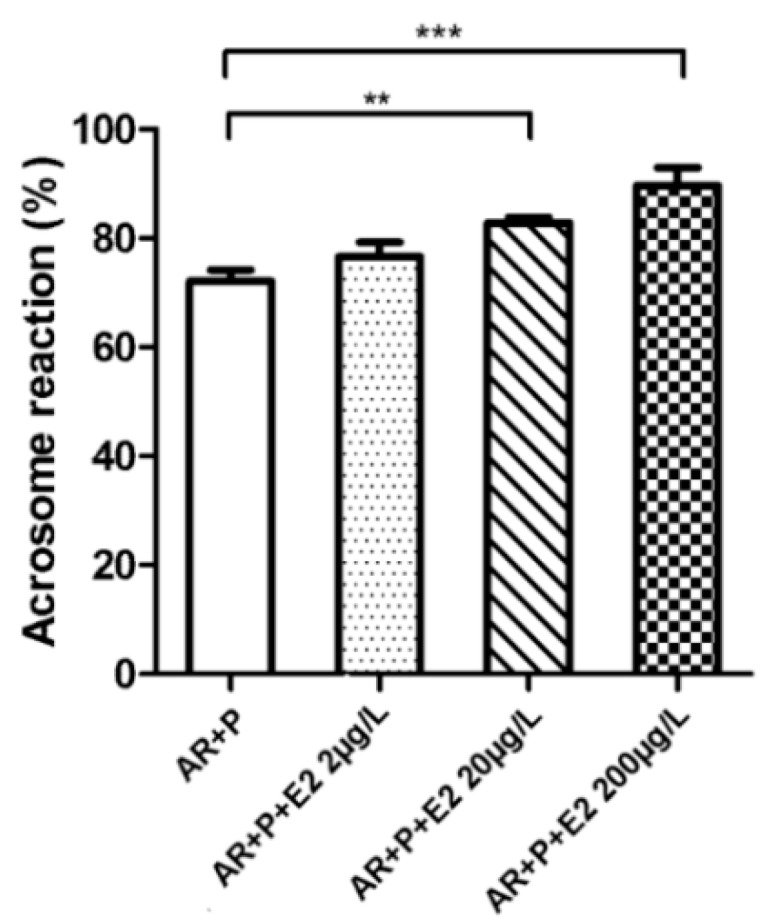
Acrosome reaction (AR) in presence of estradiol (E2) and progesterone (P). Sperm were capacitated (90 min) followed by progesterone (induced 10 μM) induced AR (90 min) in presence of E2 (AR + P + E2 2; 20 and 200 μg/L) or without E2 (control, AR + P). The results show an E2 dose-dependent increase in AR rate. A total of 200 cells were counted in six individual samples. Error bars indicate the SD. ** *p* < 0.01, *** *p* ≤ 0.001.

In order to monitor the capacitation outcome in connection to estradiol, the experiments were designed to compare the solitary effect of estradiol and its competing dose-dependent effect with progesterone during capacitation, as well as the effect of estradiol on induction of AR in relevance to progesterone after capacitation finished. The presence of estradiol from the beginning of capacitation had a stimulatory effect on the rate of spontaneous AR and was positively correlated with estradiol increasing concentration (Figure 5). In presence of both E2 and progesterone the percentage of sperm remained constant after the AR despite the expected stimulatory effect of a higher concentration of progesterone compared to estradiol. The AR remained the same between the control and experimental groups (Figure 6). The results were, however, different when sperm were exposed for 90 min to estradiol and progesterone only after completing capacitation. The AR was enhanced in the group of estradiol with progesterone. This was expected, as the progesterone concentration exceeded the concentrations of estradiol [15]; however, increasing the rate of AR was also positively correlated with increasing estradiol concentration (Figure 7), which was not the case when steroid hormones were added at the beginning of capacitation. For detailed information on methodology, please see Appendix A in Supplementary Materials.

## 3. Discussion

Estradiol concentration in follicular fluid is higher than in the reproductive female tract and comparable with our experimental concentrations [43]. Specifically, in female mammals, estradiol concentration in the ovarian fluid is at least two-fold higher compared with that of plasma [44], and it fluctuates during estrus, and between 145 and 2100 pg/mL for mouse and rat [45]. Therefore, sperm are expected to be exposed to high concentrations during certain stages of their capacitation in the female reproductive tract [43,46]. Based on these facts, the in vivo sperm exposure to estradiol is a common phenomenon. Understanding estradiol action is of importance because higher concentrations of estradiol than physiological concentrations were reported to lead to in vivo premature capacitation and a decreased fertilizing potential [12]. Sperm capacitation happens 30–90 min after introducing sperm to the female reproductive tract surroundings [3], but also depends on the estrogen concentration, which promotes a time dependent stimulatory effect and increased the protein TyrP in vitro [11] and altered the ability of sperm to undergo an induced acrosome reaction [11,13]. A recent study [5] showed that TyrP may not be essential for mouse sperm capacitation/fertilization in vivo, as an inability of tyrosine-protein kinase *Fer*^DR/DR^ mice with a kinase-inactivation mutation to promote TyrP did not result in sterility; however, their fertility outcome decreased. Our results showed that estradiol increased TyrP in capacitation sperm, the overall TyrP protein level, and the TyrP of the sperm head. It has been shown that sperm head TyrP is a crucial event prior to AR and only up to 15% of sperm reach this state [42]. Considering that decreased in vitro sperm fertility caused by absence of TyrP in mouse may be overcome simply by the interaction of sperm with the female reproductive environment [5], a hormonal player may be involved. Estradiol concentration is high in the site of sperm–egg interaction and may trigger alternative pathways or promote sperm fitness based on its inducible effect seen in the case of both sperm overall and head TyrP (Figure 3, Figure A1, Appendix B in Supplementary Materials).

In addition to the estradiol stimulatory effect on capacitation [8,9,10,11,12], the non-induced (spontaneous) AR was elevated [13]. The spontaneous acrosome reaction has been shown to be a physiological process in mouse sperm and can be completed before sperm reaches the *zona-pellucida* [47]. The acrosome reacted sperm are still able to fertilize the egg [48]; therefore, the stimulatory effect of estradiol (Figure 5) may be considered important for sperm fertilizing potential. In the presence of progesterone, the stimulatory effect of estradiol is abolished (Figure 6), which is correlated with estradiol-based suppression of progesterone-induced TyrP [14,15], hyperactivation [14,15,16], and AR [9,13]. This was not the case for spontaneous AR. The acrosome reaction is enhanced when the concentration of progesterone is dominant over estradiol, and when sperm are exposed to both steroid hormones simultaneously after completing the capacitation (Figure 7). However, spontaneous AR during capacitation remained constant (approx. 20%), despite the presence of progesterone and estradiol. When estradiol was present by itself from the beginning of capacitation, spontaneous AR was elevated, which allowed us to measure and quantify the induction effect of estradiol on sperm capacitation and deduce a potential mechanism of estradiol interaction with ERs.

To evaluate the relevance of the estradiol effect on sperm capacitation, we used CASA as powerful tool to monitor subtle specific changes in sperm motility. An additional process, called hyperactivation, occurs during sperm capacitation [49]. Hyperactivated spermatozoa are characterized by higher values of curvilinear velocity (VCL) and lateral head displacement (ALH) [35]. These changes in sperm kinematic parameters are associated with higher amplitude of flagellar beating and lower progressivity providing them greater vigour and force. Our results showed that estradiol affected VLC or ALH motility parameters depending on mouse strains, stressing the importance of considering strain-base sperm-specific motility differences. In C57BL/6NVel mice, ALH increased at 20 µg/L. In BALB/c, the parameter did not differ from the control, but there was a negative effect of the same concentration on VCL. This result agrees with previously reported sperm motility differences in these mice strains [35]. CASA algorithms calculated ALH based on the specific relationships of VCL and VAP [49]. Thus, our findings are supportive of the analysis of sperm capacitation by TyrP results, (Figure 3, Figure A1, Appendix B in Supplementary Materials) which showed estradiol had a stimulatory effect on the hyperactivation (ALH) of spermatozoa. Having validated experimental systems and relevant biological data regarding the effect of estradiol on sperm capacitation, motility, and AR, we focused on quantification of sperm-estradiol binding during capacitation by HPLC-MS/MS, and analysing sperm-estradiol interactive binding kinetics.

**Figure 8:**
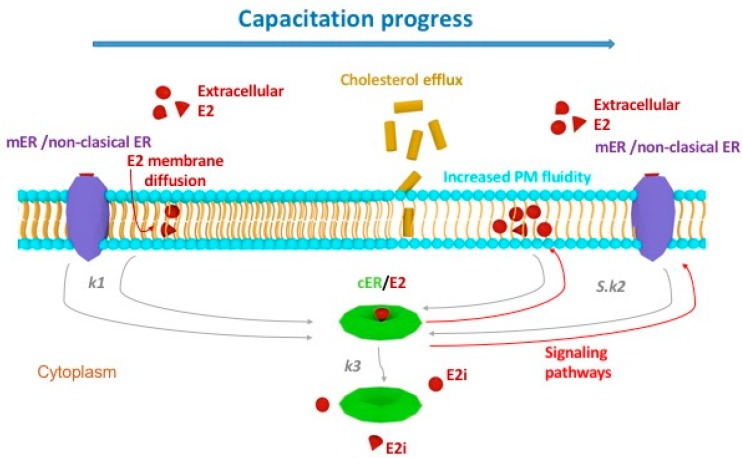
Schematic interpretation of kinetic analysis results applied on estradiol sperm action using the symbols from kinetic equations. The model depicts capacitating progress in time (blue arrow), which is represented by plasma membrane (PM) reorganization and increasing fluidity. During this process, the PM becomes more receiving for extracellular estradiol E2, which is passing via diffusion after its initial adsorption onto specific PM adsorbents, such as mERs and/or non-classical ER. Kinetic analysis of biological data suggests the formation of an adduct cER/E2 in the cytoplasm with the rate constant *k*_1_ (grey arrows), which, when formed, serves as an autocatalytic agent, signalling an increase in PM fluidity directed by *S*·*k*_2_ (red arrows). This signalling event is accompanied by the complex cER/E2 disintegration characterized by the rate constant *k*_3_ (grey arrow) and a release of estradiol E2_i_ remaining in the cytoplasm. The cERs remain internalized within the cytoplasm; however, they lose their receptivity and remain dormant.

Combining both experimental and theoretical data, we therefore propose a novel estradiol binding pattern. The schematic and simplified interpretation of the kinetic analysis results applied to data obtained from estradiol action in sperm during capacitation is summarized in Figure 8.

Capacitating sperm undergo several changes including plasma membrane reorganization, which changes its fluidity and improves reception of the extracellular estradiol E2, which passes via diffusion after its initial adsorption (controlled by Langmuir isotherm) onto specific PM adsorbents. These could be represented by mERs and/or non-classical ER. We would like to deliver a hypothesis for this mechanism, suggesting the formation of an adduct in the cytoplasm with the rate constant *k*_1_, which, when formed, serves as an autocatalytic agent, signalling an increase in PM fluidity directed by *S*·*k*_2,_ where *S* is a degree of activity. This signalling event is accompanied by the complex cER/E2 disintegration characterized by the rate constant *k*_3_ and a release of estradiol remaining in the cytoplasm (E2_i_). The cERs remain internalized within the cytoplasm; however, they lose their receptivity and remain dormant (Appendix A in Supplementary Materials).

One of the criteria for the reliability of kinetic equations is the independence of the rate constants on the concentration conditions. The determined values of the optimized overall constants *K*_2_ and *K*_3_ fulfil these conditions by well over two orders of magnitude. The correctness of the used model was further confirmed by the positions and the shapes of the minima on the *B*(*t*) curves, which agreed with the experimental values. The areas around the minima are sensitive to the rate constant values. Hewitt et al. [24] described two processes of estradiol passing through the plasma membrane, one of which is slow and the other is rapid. In our model, the slow process with the rate constant *k*_1_ occurs simultaneously with the rapid (autocatalytic) process, with the rate constant *k*_2_ multiplied by *S*.

## 4. Materials and Methods

### 4.1. Chemicals, Reagents, and Animals

Acetonitrile (ACN) Chromasolv LC/MS and deuterated β-estradiol-16,16,17-d_3_ (estradiol-d_3_) (purity 98%) were purchased from Sigma-Aldrich (Chromasolv, Schnelldorf, Germany). Ethanol (96%) was obtained from Lach-Ner (Neratovice, Czech Republic). Paraffin oil was delivered by Carl Roth (Karlsruhe, Germany). Formic acid (HCOOH) (purity 98–100%) and 17β-estradiol (estradiol) (purity 98%) were provided by Merck (Gernsheim, Germany). Commercial M2 culture media for in vitro sperm capacitation and fertilization (M7167, Sigma-Aldrich, Prague, Czech Republic) contained: HEPES (4-(2-hydroxyethyl)piperazine-1-ethanesulfonic acid), calcium chloride, magnesium sulfate, potassium chloride, potassium phosphate, sodium bicarbonate, sodium chloride, bovine serum albumin, d-glucose, pyruvic acid, and d,l-lactic acid, without penicillin and streptomycin. An experimental M2 medium, but without BSA, was prepared in the Media Preparation and Washing Units, BIOCEV (Vestec, Czech Republic) and used for simulating non-capacitating conditions [1,3]. Deionized water (Milli-Q water purification system Millipore, Billerica, MA, USA) was used in all experiments.

Two laboratory inbreed mouse strains (BALB/c and C57BL/6Nvel) were used for comparative experiments. Mice were purchased from Velaz (Prague, Czech Republic) and maintained and housed at the animal facilities of the Faculty of Science, Charles University (Prague, Czech Republic). All the animal procedures and all the experimental protocols were approved by Local Ethics Committee and carried out in strict accordance with the Animal Scientific Procedure, Art 2010, and subjected to review by this Local Ethics Committee of the Faculty of Science, Charles University, Czech Republic (accreditation no. 247732008-10001).

### 4.2. Instrumentation and Chromatographic Conditions

The HPLC equipment (Agilent Technologies, Waldbronn, Germany) including a 1290 Infinity Series LC (a quaternary pump, a degasser, a thermostatic auto sampler with a 20 μL sample loop and a column oven). Triple Quad LC/MS 6490 tandem mass spectrometer (Agilent Technologies, Waldbronn, Germany) with an electrospray ionization interface (ESI) was used for the detection. Signals were processed and data were handled with the Mass Hunter Workstation Software (Agilent Technologies, Waldbronn, Germany).

The MS-MS measurements were performed in the multiple reaction monitoring (MRM) mode using ESI ionization in positive mode (ESI (+)). Nitrogen was used as the collision nebulizing and desolvation gas. The optimized ESI (+) conditions in MRM mode for estradiol were as follows: capillary voltage, 5500 V; nebulizer pressure, 60 psi; gas temperature, 350 °C; and nitrogen flow rate 10 L/min. The *m*/*z* 255.5 to 158.9 transition (fragmentor voltage: 120 V, collision energy: 14 V) was monitored for estradiol, and the *m*/*z* 258.5 to 158.9 transition (fragmentor voltage: 120 V, collision energy: 14 V) was monitored for deuterated estradiol (estradiol-d3).

Separation was performed on a Kinetex EVO C18 column (100 × 3.0 mm i.d., particle size 2.6 µm) from Phenomenex (Torrance, CA, USA). The optimization procedure was carried out to efficiently separate of estradiol in M2 medium [50,51]. Isocratic elution at a flow rate of 0.3 mL/min with the binary solvent system, consisting of 0.1% HCOOH in H_2_O and 0.1% HCOOH in 100% ACN, 50/50 (*v*/*v*), was selected. The column temperature was held at 21 ± 0.5 °C. The sample injection was 7.5 µL. The retention time of estradiol was 3.1 min. The complex M2 medium contained inorganic and organic components, and BSA (4.0 g/L) can especially cause difficulties during the separation and detection process. The eluate was discharged from 0 to 2.5 min and passed into the MS detector for 2.5 to 4.0 min.

The stock solution of estradiol at 200 mg/L was prepared by dissolving an appropriate amount of estradiol standard in ethanol. The stock solutions at concentration of 20, 2, and 0.2 mg/L were obtained by serial dilutions with ethanol and were stored in the dark at 5 °C. Working solutions to obtain the standard points of the calibration curve were prepared by diluting the appropriate ethanolic stock solutions with capacitating M2 medium to attain the following concentrations: 1, 5, 10, 15, 20, 25, 50, 75, 100, 125, 150, 175, 200, and 225 µg/L. The internal standard (estradiol-d_3_) working solution was prepared fresh daily in ethanol at a concentration of 250 µg/L. We added 100 µL of this solution to each calibration standard solution to attain the final concentration of 25 µg/L.

Under optimized separation and detection conditions, the calibration curve for estradiol was measured in the concentration range of 1–225 µg/L and the analyte was tested within a linearity range from limit of quantitation (LOQ) to 225 µg/L. Each measurement of the peak area (peak height) was performed in 5 replicates and the results of the linear regression for the peak area ratio of the analyte to the internal standard versus concentration are listed in Table A3, Appendix B in Supplementary Materials. A satisfactory fit between the experimental points and linear calibration curve was observed. The peak height-concentration dependence was treated by linear regression to determine the limit of detection (LOD) and LOQ, as 3× and 10× noise levels, respectively. The values obtained for LOD and LOQ are presented in Table A3, Appendix B in Supplementary Materials.

### 4.3. Capacitation of Mouse Sperm In Vitro and Sample Preparation for HPLC-MS/MS Analysis

For the in vitro realization of capacitation, 35- mm Petri dishes obtained from Corning (Corning, NY, USA) were used. An Olympus CX 21 light inverted microscope and Olympus epifluorescent microscope were supplied by Olympus (Prague, Czech Republic). The NB-203 incubator was purchased from N-BIOTEK (Bucheon, Korea). For in vitro cultivation of sperm, incubator Telstar Bio-IIA and laminar box-BioTek from N-BIOTEK (Bucheon, Korea) were used.

The physiological estradiol concentrations relevant to the reproductive system are known in rats, mice, and humans. The concentrations vary from 250 pg/mL in the rete testis fluid in rats [44,52] to 1 ng/mL in the spermatic vein of men [53], and fluctuates in plasma during estrous between 145–2100 pg/mL in rat and mouse [45] and 90–400 pg/mL in woman [54]. In the ovarian fluid, the concentration is at least two-fold higher than in plasma [44,52]. Due to this wide concentration range, the in vitro system, and the detection limit of HPLC/MS-MS, we used the working experimental estradiol solutions with dilutions of 2, 20 and 200 µg/L. These concentrations were prepared by diluting the ethanolic stock solution of estradiol (200, 20 or 2 mg/L) with capacitating M2 medium (Sigma-Aldrich, Prague, Czech Republic) or non-capacitating medium without BSA (Media Preparation and Washing Units, BIOCEV, Vestec, Czech Republic) to attain the required experimental concentration according to the following scheme: 1 µL of appropriate ethanolic solution was diluted into 1 mL of capacitating M2 or non-capacitating medium to minimize the amount of ethanol in biological sample. Then, 100 µL of this solution was placed into each of the Petri dishes and covered with 1 mL paraffin oil. All procedures were performed in a sterile laminar box. Prepared Petri dishes were then placed for 60 min to incubate at 37 °C in 5% CO_2_ in air.

The biological sample was prepared in three parallel sets and each set represented sperm collecting from one individual. Spermatozoa, which were recovered from the distal region of the *cauda epididymidis*, were placed in capacitating M2 or non-capacitating medium and left in an incubator for 10 min at 37 °C under 5% CO_2_ to relax sperm. After that, the concentration of stock sperm in the medium was adjusted to 5 × 10^6^ sperm/mL. The motility and viability of the sperm population was checked throughout the experiment using a light inverted microscope with a thermostatically controlled stage at 37 °C. It remained unchanged during experimental time and conditions when compared with the stock solution.

There were four groups labelled as follows: (1) M (control, sperm in capacitating medium), (2) M + estradiol (capacitating medium with addition of estradiol: 200, 20, or 2 µg/L), (3) M without BSA (non-capacitating medium), and (4) M without BSA with estradiol (non-capacitating medium with addition of estradiol: 200 µg/L). Samples were prepared in Petri dishes according to the following scheme: after 60 min incubation of 100 µL of 200, 20, or 2 µg/L solution of estradiol in media, 5 µL of sperm stock solution was added. Each time, 6 Petri dishes were used, each one containing 105 µL of sample volume covered with 1 mL paraffin oil. Control samples, using only capacitating M2 or non-capacitating medium without the addition of estradiol, were run in parallel in each experiment. Spermatozoa were incubated (37 °C, 5% CO_2_) for up to 3 h. At half hour intervals (0, 30, 60, 90, 120, 150, and 180 min after adding sperm) samples were collected. The sample solutions from all Petri dishes (for a given time) were placed into one micro tube to centrifugate for 10 min at 12,000 rpm to remove the spermatozoa. After centrifugation, 450 µL of the supernatant was placed into a vial for HPLC-MS/MS analysis. To avoid potential systematic errors during sample preparation (partial evaporation of samples during incubation, differences in collection of supernatant after centrifugation, etc.), reference samples (blanks) were prepared collaterally with the samples described above, but no spermatozoa were added into the incubated estradiol solution. To check the correctness of HPLC-MS/MS analysis, 50 µL of internal standard working solution at a concentration of 250 µg/L was added into each sample before the measurement. Each sample was measured in 5 replicates and the mean value was calculated.

The matrix effect was measured for all tested estradiol concentrations (2, 20, and 200 µg/L) by comparing the peak response of (1) the supernatant prepared by addition of sperm in the medium and after centrifugation spiked with estradiol and with (2) the sample prepared by addition of estradiol directly into the medium and no matrix effect was observed.

### 4.4. SDS-PAGE with Immunoblotting

SDS (Sodium dodecyl sulfate) electrophoresis and immunoblotting were used for the TyrP assessment, carried out using protocols based on standard methods [55,56]. Suspension of non-capacitated sperm from a sperm stock released from the cauda epididymis was used. Sperm samples were collected at 0, 60, and 90 min of capacitation in vitro, diluted with PBS, and a final concentration of 5 × 10^6^ sperm/mL was ascertained using a Bürker chamber (Sigma-Aldrich, Prague, Czech Republic). Sperm pellets were re-suspended in an equal volume of SDS-PAGE (sodium dodecylsulphate-polyacrylamide gel-electrophoresis) reduced sample buffer and heated at 97 °C for 3 min. Samples containing protein equivalent to 5 × 10^6^ capacitated sperm per mL were run on a 5% stacking and 10% running SDS polyacrylamide gel using Precision Plus Protein™ Dual Color Standards (Bio-Rad, München, Germany) as molecular weight markers. After transferring protein onto a nitrocellulose membrane, nonspecific sites were blocked with PBS blocking solution (5% skimmed milk and 0.05% Tween 20). Proteins phosphorylated on tyrosine residues were identified by the primary MAB (monoclonal antibody) anti-phosphotyrosine P-Tyr-01 (Exbio, Vestec, Czech Republic) diluted 1:300, followed by a peroxidase goat anti-mouse IgG (Immunoglobulin G) secondary antibody (Sigma-Aldrich, Prague, Czech Republic) diluted 1:5000. β tubulin was detected by anti-β tubulin primary antibody ab15568 (Abcam, Cambridge, MA, USA), diluted 1:200, followed by peroxidase goat anti-rabbit IgG secondary antibody 1706515 (Bio-Rad, München, Germany) diluted 1:3000. Protein staining was visualised by chemiluminescence Super Signal West Dura (Thermo Fischer Scientific, Prague, Czech Republic). These experiments were performed four times with similar results. Representative results are shown.

### 4.5. Statistical Analysis

Experimental data were analyzed using STATISTICA 6.0 (Statsoft, Prague, Czech Republic) and GraphPad Prism 5.04 (GraphPad Software Inc., La Jolla, CA, USA). The differences between the control and experimental groups in the number of TyrP positive sperm heads were analyzed by KW-ANOVA (Kruskal–Wallis test), and post-hoc analysis was performed by Dunn’s comparisons: * *p* < 0.05, ** *p* < 0.01, *** *p* < 0.001.

We used R (v 3.4.0) [57] to evaluate the general effects of estradiol concentrations on sperm performance in two mouse strains (BALB/c and C57BL/6Nvel). To avoid pseudo replication, analyses were based on mean trait values measured on each male mouse in respective time and estradiol concentration, rather than on performance of each sperm cell. All three measures of sperm velocity—the strait velocity (VSL), curvilinear velocity (VCL), and the average path velocity (VAP)—were strongly correlated with each other (Pearson product-moment correlation coefficients of 0.97 in all comparisons). As the medium did not contain any component to guide the spermatozoa in one direction, we used curvilinear velocity (VCL) rather than the others measures as our measurement of sperm swimming speed [58,59]. There was only a moderate association between the lateral head displacement (ALH) and three measures of sperm velocity (Pearson product-moment correlation coefficients ranged between 0.39 and 0.58). Therefore, VCL and lateral head displacement (ALH) were analyzed separately. We used the lmer() function in the lme4 package [59] for fitting linear mixed effect model with either sperm motility (VCL) or the amplitude of ALH as the dependent variable and estrogen concentration (categorical variable with four levels: control, 2, 20, and 200 μg/L), strain identity, and their two-way interaction as fixed effects. To control for repeated observation within male mice over time, male identity was included as random intercept and time of measurement (0, 30, 60, and 120 min) as random slope. To obtain *p*-values, we performed likelihood ratio tests comparing models with and without a specific fixed effect.

## 5. Conclusions

Based on presented data, estradiol has a stimulatory effect on protein tyrosine phosphorylation, lateral head displacement, and spontaneous acrosome reaction during in vitro mouse sperm capacitation. The level of estradiol available for mouse spermatozoa during capacitation in vitro was quantified by HPLC-MS/MS and data were subjected to kinetic analysis. The proposed kinetic model explains the crosstalk between estradiol and both receptor and non-receptor pathways, suggesting an autocatalytic signalling pattern as a novel mechanism utilized by sperm prior to fertilization. There is a potential in this new analytical-biological approach for understanding the physiological mechanism of steroid hormones, including estradiol, not only in reproductive biology, but also in somatic cell signalling events, both physiological and pathological, targeting mainly cancer cell biology research.

## Figures and Tables

**Table 1:** Relative concentrations of total unbound estradiol (*B*_t_) calculated from the measured time-dependent concentrations of total unbound estradiol (*C*) (Figure 1A–C) obtained during capacitation for three tested estradiol concentrations and two inbreed mouse strains; mean ± standard error of the mean.

Capacitation Time (min)	*B*_t_					
	200 µg/L		20 µg/L		2 µg/L	
	BALB/c	C57BL/6N	BALB/c	C57BL/6N	BALB/c	C57BL/6N
0	1.000 ± 0.003	1.000 ± 0.003	1.000 ± 0.028	1.000 ± 0.023	1.000 ± 0.050	1.000 ± 0.070
30	0.965 ± 0.005	0.944 ± 0.004	0.965 ± 0.033	0.958 ± 0.024	0.987 ± 0.055	0.988 ± 0.073
60	0.983 ± 0.004	0.952 ± 0.003	0.919 ± 0.026	0.938 ± 0.024	0.969 ± 0.061	0.982 ± 0.079
90	0.989 ± 0.005	0.968 ± 0.005	0.874 ± 0.031	0.904 ± 0.025	0.938 ± 0.055	0.923 ± 0.071
120	0.995 ± 0.004	0.979 ± 0.004	0.886 ± 0.028	0.921 ± 0.025	0.838 ± 0.046	0.858 ± 0.069
150	0.997 ± 0.004	0.984 ± 0.005	0.914 ± 0.032	0.929 ± 0.023	0.751 ± 0.062	0.826 ± 0.066
180	0.998 ± 0.005	0.989 ± 0.005	0.958 ± 0.028	0.956 ± 0.022	0.773 ± 0.058	0.947 ± 0.074

**Table 2:** Calculated constants for three tested estradiol concentrations and two inbreed mouse strains, where *D* is dilution factor, *n* is molar ratio, and *K*_2_ and *K*_3_ are overall rate constants.

Constants	200 µg/L		20 µg/L		2 µg/L	
	BALB/c	C57BL/6N	BALB/c	C57BL/6N	BALB/c	C57BL/6N
*D*	1	1	0.1	0.1	0.01	0.01
*n*	12	12	1.2	1.2	0.12	0.12
*K*_2_	4.0	2.0	4.5	4.0	4.5	5.5
*K*_3_	3.0	1.3	4.3	6.0	3.5	6.5

## Supplementary Materials

The following are available online at http://www.mdpi.com/1422-0067/19/12/4011/s1. Supplementary Appendix A: Materials and Methods containing: Kinetic analysis with detailed description of mathematical solutions, Acrosome reaction, Immunofluorescent detection of sperm heads protein TyrP and Analysis of sperm motility. Supplementary Appendix B: Table A1: The mean concentrations (*C*, μg/L) of total unbound estradiol and their standard deviations obtained by HPLC-MS/MS, Table A2: Results of linear mixed-effect models involving curvilinear velocity (VCL) or the amplitude of lateral head displacement (ALH), Table A3: Parameters of the calibration curve, Figure A1: Sperm head Tyrosine phosphorylation.
