# Supplementary material for: New Insight into Sperm Capacitation: A Novel Mechanism of 17β-Estradiol Signalling
Source: Int J Mol Sci. 2018 Dec 12;19(12):4011. doi: 10.3390/ijms19124011 (PMC6321110; doi:10.3390/ijms19124011)
Supplement: Supplementary file 1 [file ijms-19-04011-s001.pdf]

Supplementary Materials

New insight into sperm capacitation: A novel mechanism of 17β-estradiol signaling

Appendix A

Material and Methods

Kinetic analysis

In the search for various kinetic models it was found that, for the agreement between the curves obtained by fitting through the experimental points and the theoretical calculated curves, it is necessary to assume that the first step is adsorption of estradiol onto the surface of the sperm controlled by Langmuir isotherm. Other models (without adsorption) lead to completely different results.

The following model in which the symbols of the species also correspond to their molar concentrations was used for the autocatalytic process. The Langmuir adsorption of estradiol onto the surface of sperm PM first occurs with a starting estradiol concentration ($E2_0$), and the surface of PM at these sites (number n_a) becomes more accessible. It holds for n_a that: $n_a = \frac{zw(E2_0)}{1+w(E2_0)}$ where z is the maximum number of adsorption sites ((mER) or membrane non-estrogen receptors) and w is the adsorption coefficient. Well below the saturation point ($1 > w\ (E2_0)$) it holds that $n_a = z\ w\ (E2_0)$. At sites n_a the externally present estradiol (concentration of extracellular estradiol at the time > 0, ($E2$)) reacts with the sperm membrane receptors leading to increased permeability of PM, through which estradiol molecules are transported within the cytoplasm to (cER) and forming the adduct (($E2$)/(cER)). This is connected with a further increase in the PM permeability. The primary penetration of PM corresponds to the following kinetic product, where k_1 is the rate constant corresponding to the formation of aduct:

$$k_1\, n_a\, (E2)\, (cER) \tag{1a}$$

The gradual growth of adducts (($E2$)/(cER)) in the cytoplasm leads to ever increasing permeability of PM (signalling). Activity Γ increases and is proportional to the consumed estradiol $\Gamma = k\,((E2_0) - (E2))$ so that, at the end of the reaction, $\Gamma_\infty = k\,(E2_0)$, the degree of activity S can be defined as: $S = \frac{\Gamma}{\Gamma_\infty} = \frac{(E2_0)-(E2)}{(E2_0)}$

The formation of the adduct (($E2$)/(cER)) is thus enriched by the autocatalytic reaction, with the corresponding kinetic product:

$$k_2\, (E2)\, (cER)\, S \tag{1b}$$

where k_2 is the rate constant corresponding to the elevated degree of permeability of PM through the formation of adduct (($E2$)/(cER)) in the cytoplasm. The formed adduct (($E2$)/(cER)) is not stable and decomposes with the formation of internal estradiol (i.e.

inside the cytoplasm, ($E2_i$)) and this kinetic equation corresponds to the kinetic product:

$$k_3\ ((E2)/(cER)) \tag{1c}$$

where k_3 is the rate constant corresponding to the decomposition ($(E2)/cER$)) of the adduct.
Thus, using Eqs.1a – c, we can write for the overall rates of the individual steps:

$$\frac{-d(E2)}{dt} = k_1 z\, w\, (E2_0)\, (E2)\, (cER) + k_2 S\, (E2)\, (cER) \tag{2a}$$

$$\frac{-d(cER)}{dt} = k_1 z\, w\, (E2_0)\, (E2)\, (cER) + k_2 S\, (E2)\, (cER) \tag{2b}$$

$$\frac{d((E2)/(cER))}{dt} = k_1 z\, w\, (E2_0)(E2)\, (cER) + k_2 S\, (E2)\, (cER) - k_3\, ((E2)/(cER)) \tag{2c}$$

$$\frac{d(E2_i)}{dt} = k_3\, ((E2)/(cER)) \tag{2d}$$

with the following initial conditions:

$(E2)_{(t=0)} = (E2_0)$, $((E2)/(cER))_{(t=0)} = 0$, $(E2_i)_{(t=0)} = 0$, $(cER)_{(t=0)} = (cER_0)$

It follows from Eqs.2a, b that:

$$\frac{d(E2)}{dt} = \frac{d(cER)}{dt}, \text{ and after integration: } (E2) - (E2_0) = (cER) - (cER_0) \tag{3a}$$

adding Eqs. 2a, c, d yields: $\frac{d(E2)}{dt} + \frac{d((E2)/((cER))}{dt} + \frac{d(E2_i)}{dt} = 0$ and, after integration:

$$(E2) - (E2_0) + ((E2)/(cER)) + (E2_i) = 0 \tag{3b}$$

In the next step we express from Eq.3a: $(cER) = (E2) - (E2_0) + (cER_0)$, substituting into Eqs. 2a, c where simultaneously we write S as $\frac{(E2_0)-(E2)}{(E2_0)}$ and rearrange:

$$\frac{-d(E2)}{dt} = (E2)((E2) - (E2_0) + (cER_0))(k_1 z\, w\, (E2_0) + k_2\left(1 - \frac{(E2)}{(E2_0)}\right)) \tag{4a}$$

$$\frac{d((E2)/(cER))}{dt} = (E2)((E2) - (E2_0) + (cER_0))(k_1 z\, w\, (E2_0) + k_2\left(1 - \frac{(E2)}{(E2_0)}\right)) - k_3((E2)/(cER)) \tag{4b}$$

$$\frac{d(E2_i)}{dt} = k_3((E2)/(cER)) \tag{4c}$$

Internal ($E2_i$) is formed after decomposition (($E2$)/(cER)) and then it follows from Eq.3b that:

$$(E2_i) = (E2_0) - (E2) - ((E2)/(cER)) \quad (4d)$$

In the above-described measuring method, after completion of the reaction by intense centrifugation, the centrifugate contains both internal $(E2_i)$ and extracellular $(E2)$ estradiol and the sperm together with the adduct $((E2)/(cER))$ remain in the sediment. The measured concentration (C) is proportional to the total unbound estradiol content: $k\,((E2) + (E2_i))$ and Eq.4d yields its theoretical value:

$$C = k\big((E2_0) - ((E2)/(cER)\big), \quad C_0 = k\,(E2_0) \quad (5a)$$

In order to simplify the set of Eqs. 4a, b as much as possible, minimize the number of variables and eliminate unknown values of constants z, w and (cER_0), the relative concentrations will now be introduced: $\varepsilon = \frac{(E2)}{(E2_0)}$, $\alpha = \frac{((E2)/(cER))}{(E2_0)}$, the molar ratio $n = \frac{(E2_0)}{(cER_0)}$, dimensionless time $\tau = t\,(cER_0)\,k_1 z\,w\,(E2_{01}) = t\,(cER_0)\,K_1$, (so that $K_1 = k_1 z\,w\,(E2_{01})$) and also constants $K_2 = \frac{k_2}{K_1}$, $K_3 = \frac{k_3}{(cER_0)K_1}$ and fraction $D = \frac{(E2_0')}{(E2_{01})}$, where it is best to take the highest added estradiol concentration for $(E2_{01})$ and $(E2_0')$ is the currently selected initial estradiol concentration, so that $\frac{(E2_0')}{(E2_{01})}$ can assume values of 1, 0.1 or 0.01. Thus, the D values are fixed by dilution.

The $B(t)$ value for the sperm of the BALB/c and C57BL/6Nvel strains of mice can then be expressed as:

$$B(t) = 1 - \frac{((E2)/(cER))}{(E2_0)}, \text{ thus } B(t) = 1 - \alpha \quad (5b)$$

The set of differential Eqs.4a – c then changes to the form:

$$\frac{-d\varepsilon}{d\tau} = \varepsilon\,(n(\varepsilon - 1) + 1)\,(D + K_2(1 - \varepsilon)) \quad (6a)$$

$$\frac{d\alpha}{d\tau} = \varepsilon\,(n(\varepsilon - 1) + 1)\,\big(D + K_2(1 - \varepsilon)\big) - K_3\alpha \quad (6b)$$

with initial conditions $\varepsilon_{(t=\infty)} = 1$ and $\alpha_{(t=0)} = 0$.

The reaction is theoretically terminated for $n < 1$, when $\varepsilon = 0$, $\alpha = 0$ and thus $B_{t\infty} = 1$. For $n > 1$ the reaction is terminated for $(cER) = 0$, or $n(\varepsilon - 1) + 1 = 0$, so that $\varepsilon_{(t=\infty)} = 1 - \frac{1}{n}$. According to Eq.5b, it also holds for $\alpha = 0$ that $B_{t\infty} = 1$. Thus, all the curves end at limiting value 1. Equations 6a, b are solved by the fourth-order Runge-Kutta method with step of $h = 10^{-4}$. The actual time t is one hundred times greater than the calculated time τ and the results of the calculations are given in Table 2. Values D were determined by diluting the highest employed estradiol concentrations (200μg/L), corresponding to a value of $D = 1$. The molar ratio n was estimated from the shape of curve $B(t)$ for dilution of $D = 1$. The other dilutions are given by a tenth and hundredth

of the original value. Constants K_2 and K_3 were obtained by optimization (searching the minimum of absolute values of the difference between theoretical and experimentally obtained B_t values).

Acrosome reaction

Petri dishes were prepared containing either 100μL of M2 medium under paraffin oil (control) or 100μL of M2 medium containing concentrations of estradiol (200, 20 and 2μg/L) and capacitated for 90min as stated above in Capacitation method. The experimental dishes containing individual estradiol concentrations had the following set up: 1: estradiol alone was present in the M2 medium from the beginning of sperm capacitation (time 0); 2: progesterone (10μM, 3144μg/L) was added to the medium together with estradiol at the beginning of capacitation (time 0); 3: Both estradiol and progesterone were added after 90min of capacitation and sperm were incubated for an additional 90min.

Sperm smears were prepared for every *in vitro* experiment stated above. Sperm were washed twice in PBS, smeared onto glass slides, air dried and fixed with 3.7% formaldehyde in PBS (pH 7.34) at room temperature for 10min, followed by washing in PBS. PNA lectin (Molecular Probes) was added at the concentration of 2.5mM in PBS. After washing, the slides were mounted into Vectashield mounting medium with DAPI (Vector Lab., Burlingame, CA, USA). The samples were examined with an Olympus IX81 fluorescent microscope. The rate of AR was monitored in 200 sperm in six individual experiments for control and each estradiol concentration and experimental groups.

Immunofluorescent detection of sperm heads protein TyrP

Sperm samples were spread on microscope slides. After air-drying, sperm were fixed with 3.7% formaldehyde in PBS (pH 7.34) at room temperature for 10min. Slides were washed with PBS which was followed by immunofluorescent staining. Sperm were blocked with 10% BSA in PBS for 1h and incubated with primary MAB anti-phosphotyrosine P-Tyr-01 (Exbio, Prague, Czech Republic) diluted 1:500 in 1% BSA in PBS over night at 4°C, followed by Alexa Fluor 488 donkey anti-mouse IgG (Molecular Probes, Prague, Czech Republic) secondary antibody 1:1000 in PBS for 1h. Slides were mounted in Vectashield mounting medium with DAPI (Vector Laboratories, Burlingame, CA, USA). Slides were examined with an epifluorescent microscope. For every experiment, we collected sperm data from eight mice. The positive or negative signal was evaluated from a total of 200 spermatozoa on every slide. In each group, at least two samples were analyzed. Data were analyzed statistically.

Analysis of sperm motility

Sperm motility parameters were analysed in experimental groups 1: M (control, sperm in capacitating medium) and 2: M + estradiol (capacitating medium with addition of estradiol - 200, 20 or 2μg/L). Preparation of samples was done in Petri dishes according to the following scheme: after 60min incubation of 100μL of 200, 20 or 2μg/L solution of estradiol in media, sperm stock solution was added to obtain a final concentration of $5x10^6$ sperm/mL. Each time 4 Petri dishes were used, each one

contained 105μL of sample volume covered with 1mL of paraffin oil. Spermatozoa were incubated (37°C, 5% CO_2) for up to 2h. At intervals (0, 30, 60, 90, 120 min after adding sperm) samples were collected. CASA Hamilton Thorne CEROS (Hamilton Thorne, Inc., USA) was used for sperm motility measurement. The system comprises of a computer, microscope (CX41, Olympus) with negative phase contrast, heating table and a camera (UI-1540-C, Olympus). Sperm tracks were captured in minimally 10 fields at 37°C with a10x negative phase contrast objective (1). Afterwards, recordings were controlled in playback mode to avoid objects, which were not spermatozoa.

Sperm suspensions were gently homogenized before analysis. For each motility measurement 30μl of sample was loaded into a chamber (100μm depth) of pre-warmed Leja Slide (Netherlands). To minimize an effect of flowing liquid, excess was removed from outside by cellulose tissue as recommended by the manufacturer. Recording was performed in the entire area of the chamber, but recorded fields were located out of edges to avoid Segre-Silberberg effect (2). Track and kinematics parameters were recorded for individual spermatozoa. Following kinematic parameters were analysed: curvilinear velocity (VCL, μm/s), average path velocity (VAP, μm/s), straight line velocity (VSL, μm/s) and amplitude of lateral head displacement (ALH, μm).

Reference

1. Albrechtová, J.; Albrecht, T.; Baird, S.J.E.; Macholán, M.; Rudolfsen, G.; Munclinger, P.; Tucker, P.J.; Piálek, J. Sperm-related phenotypes implicated in both maintenance and breakdown of a natural species barrier in the house mouse. *Proc. Roy. Soc.* **2012,** *B 279*, 4803-4810. doi: 10.1098/rspb.2012.1802
2. Douglas-Hamilton, D.H.; Smith, N.G.; Kuster, C.E.; Vermeiden, J.P.W.; Althouse, G.C. Particle Distribution in Low-Volume Capillary-Loaded Chambers. *J Androl* **2005**, *26*, 107-114. doi: 10.1002/j.1939-4640.2005.tb02879.x

Appendix B

Tables and Figures

Table A1. The mean concentrations (*C*, μg/L) of total unbound estradiol and their standard deviations ($n' = 3$) obtained by HPLC-MS/MS for three tested concentrations (200, 20 and 2 μg/L); the mean values were calculated as the average of the mean values (each sample was measured in 5 replicates) obtained for three parallel sets.

**200 μg/L**			
**Capacitation time (min)**	**BALB/c**	**C57BL/6Nvel**	**Blank**
0	196.00 ± 0.80	195.50 ± 0.73	195.70 ± 0.40
30	189.14 ± 0.75	184.53 ± 0.68	196.00 ± 0.60
60	192.67 ± 0.72	186.20 ± 0.30	195.70 ± 0.20
90	193.91 ± 0.86	189.93 ± 0.82	196.00 ± 0.70
120	195.10 ± 0.65	191.50 ± 0.71	196.00 ± 0.50
150	195.41 ± 0.50	192.37 ± 0.77	196.40 ± 0.10
180	195.61 ± 0.70	193.36 ± 0.80	196.10 ± 0.60

**20 μg/L**			
**Capacitation time (min)**	**BALB/c**	**C57BL/6Nvel**	**Blank**
0	17.90 ± 0.60	18.10 ± 0.50	18.30 ± 0.20
30	17.28 ± 0.40	17.39 ± 0.20	18.25 ± 0.15
60	16.45 ± 0.10	16.97 ± 0.20	18.40 ± 0.25
90	15.64 ± 0.40	16.36 ± 0.30	18.30 ± 0.10
120	15.86 ± 0.30	16.67 ± 0.30	18.35 ± 0.25
150	16.36 ± 0.40	16.92 ± 0.20	18.20 ± 0.30
180	16.96 ± 0.20	17.30 ± 0.10	18.25 ± 0.29

**2 μg/L**			
**Capacitation time (min)**	**BALB/c**	**C57BL/6Nvel**	**Blank**
0	1.60 ± 0.10	1.69 ± 0.06	1.71 ± 0.05
30	1.58 ± 0.05	1.67 ± 0.05	1.70 ± 0.04
60	1.55 ± 0.07	1.64 ± 0.07	1.72 ± 0.04
90	1.50 ± 0.06	1.56 ± 0.06	1.71 ± 0.04
120	1.34 ± 0.09	1.45 ± 0.08	1.69 ± 0.05
150	1.20 ± 0.10	1.38 ± 0.05	1.71 ± 0.04
180	1.24 ± 0.09	1.60 ± 0.07	1.70 ± 0.03

Table A2. Results of linear mixed-effect models involving curvilinear velocity (VCL) or the amplitude of lateral head displacement (ALH) as dependent variables, and estradiol concentration as fixed explanatory variable in two inbred mouse strains. Male identity ($n = 3$ in BALB/c and 4 in C57BL/6N) was included as random intercept, while time of recording (0s, 30s, 60s and 120s) was included as random slope. Treatment contrasts (estradiol concentration = 0) are shown, with control group mean values as intercepts; values for other groups are expressed as differences (and associated standard errors) between the mean of each group and mean of the respective control group.

	**BALB/c**		**C57BL/6N**	
**Estradiol concentrations**	Estimate [SE]	*t*-value	Estimate [SE]	*t*-value
**VLC**				
control (intercept)	82.88 [3.17]	26.13	107.99 [4.41]	24.47
**2μg/L**	-12.78 [3.45]	-3.71	-0.14 [2.58]	-0.05
**20μg/L**	-7.98 [3.45]	-2.35	-2.61 [2.58]	-1.01
**200μg/L**	-1.52 [3.45]	-0.44	0.08 [2.58]	0.03
**ALH**				
control (intercept)	12.04 [0.34]	35.95	12.41 [0.20]	61.15
**2μg/L**	-0.38 [0.47]	-0.78	0.43 [0.25]	1.69
**20μg/L**	-0.85 [0.47]	-1.79	0.74 [0.25]	2.89
**200μg/L**	0.08 [0.47]	0.17	0.40 [0.25]	1.55

Table A3. Parameters of the calibration curve (standard deviations in parentheses), limit of detection (LOD) and quantitation (LOQ) for estradiol in M2 capacitating medium, a.u – arbitrary unit.

Compound	**Slope (L/μg a.u.·s)**	**Intercept (a.u.·s)**	**Correlation coefficient**	**LOD (μg/L)**	**LOQ (μg/L)**
Estradiol	0.0152 (0.0033)	0.0704 (0.0091)	0.9955	0.3	1.1

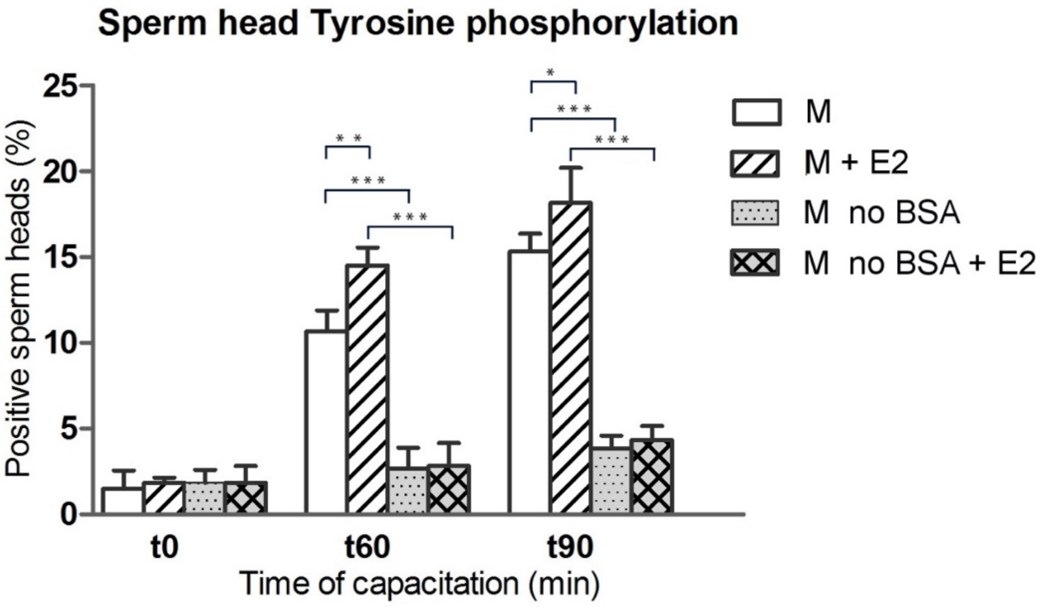

Figure A1. Sperm head Tyrosine phosphorylation. Number of positive sperm heads for protein TyrP after 0, 60 and 90min of capacitation in presence of estradiol (E2) under capacitating (capacitating M2 medium: M/M+E2) and non-capacitating (BSA absence in the medium: M no BSA/M no MSA+E2) conditions evaluated by immunofluorescent staining. Capacitation progress was measured by anti-TyrP antibody. *P<0.05, **P<0.01, ***P<0.001.

Reference

1. Albrechtová, J.; Albrecht, T.; Baird, S.J.E.; Macholán, M.; Rudolfsen, G.; Munclinger, P.; Tucker, P.J.; Piálek, J. Sperm-related phenotypes implicated in both maintenance and breakdown of a natural species barrier in the house mouse. *Proc. Roy. Soc.* **2012,** *B 279*, 4803-4810. doi: 10.1098/rspb.2012.1802
2. Douglas-Hamilton, D.H.; Smith, N.G.; Kuster, C.E.; Vermeiden, J.P.W.; Althouse, G.C. Particle Distribution in Low-Volume Capillary-Loaded Chambers. *J Androl* **2005**, *26*, 107-114. doi: 10.1002/j.1939-4640.2005.tb02879.x
